# Impact of nutritional guidance on various clinical parameters in patients with moderate obesity: A retrospective study

**DOI:** 10.3389/fnut.2023.1138685

**Published:** 2023-03-16

**Authors:** Kayoko Oda, Takatoshi Anno, Nozomi Ogawa, Yukiko Kimura, Fumiko Kawasaki, Kohei Kaku, Hideaki Kaneto, Mutsuko Takemasa, Miyori Sasano

**Affiliations:** ^1^Department of Nutrition, Kawasaki Medical School General Medical Center, Okayama, Japan; ^2^Department of Clinical Nutrition, Kawasaki University of Medical Welfare, Kurashiki, Japan; ^3^Department of General Internal Medicine 1, Kawasaki Medical School, Okayama, Japan; ^4^Department of Diabetes, Metabolism and Endocrinology, Kawasaki Medical School, Kurashiki, Japan

**Keywords:** obesity, nutritional guidance, BMI, metabolism, registered dietitian

## Abstract

**Context:**

This study aims to investigate whether there is adequate provision of nutritional guidance through interventions by registered dietitians, especially for patients with moderate obesity. This is particularly important as such interventions may prove to be more effective for Japanese patients.

**Methods:**

In Japan, since there is a system of nutritional guidance with a registered dietitian for patients with a BMI over 30 kg/m^2^, we recruited 636 patients with obesity who had a BMI over 30 kg/m^2^ admitted to the Kawasaki Medical School General Medical Center between April 2018 and March 2020 through a review of their medical records. Second, we recruited 153 patients who underwent a blood examination before receiving nutritional guidance and at least one time every 3 to 6 months thereafter after receiving it. We aimed to evaluate whether continued nutritional guidance and follow-up interventions for patients with obesity were effective. We compared the BMI and metabolic markers of the patients who received nutritional guidance from a registered dietitian against those who did not.

**Results:**

A total of 636 patients with obesity who have a BMI over 30 kg/m^2^ were included in this study. A total of 164 patients with obesity received nutritional guidance from a registered dietitian at least one time, but 472 patients did not. Most interventions on nutritional guidance conducted by a registered dietitian were ordered from internal medicine (81.1%). However, internal medicine was the most common department that did not perform these interventions; however, less than half of the (49.2%) received them. In the second analysis, we compared two groups of patients with obesity. The first group (*n* = 70) who underwent blood examinations received nutritional guidance from a registered dietitian, while the second group (*n* = 54) did not receive such guidance. We found that there was no significant difference in body weight and BMI between the two groups of patients. We observed a significant decrease in dyslipidemia-associated metabolic markers among the patients who received nutritional guidance compared to those who did not [total cholesterol, −9.7 ± 29.3 vs. 2.3 ± 22.0 mg/dL (*p* = 0.0208); low-density lipoprotein cholesterol, −10.4 ± 30.5 vs. −2.0 ± 51.0 mg/dL (*p* = 0.0147), respectively]. Other metabolic markers also tended to decrease, although they did not reach statistical significance.

**Conclusion:**

It is rare for patients with only obesity to receive nutritional guidance. However, when nutritional guidance from a registered dietitian is provided, improvements in BMI and metabolic parameters can be expected.

## 1. Introduction

Lifestyles and dietary changes have increased the number of patients with obesity in Japan and in other countries (1). The Japanese have accumulated visceral rather than subcutaneous fat, and even moderate obesity can cause various lifestyle-related diseases (2). According to the World Health Organization, overweight and obesity are defined when a body mass index (BMI) ≥ 25 and ≥ 30 kg/m^2^, respectively (https://www.who.int/news-room/fact-sheets/detail/obesity-and-overweight). However, in Japan, the threshold for defining obesity is set at a BMI of over 25 kg/m^2^. This is due to the higher risk of obesity-related complications observed even in patients with a lower BMI compared to the West (3). Diet therapy with weight loss likely decreases various obesity-related diseases and other lifestyle-related diseases, such as metabolic syndrome (4). Reducing the amount of visceral fat in the body while improving metabolic syndrome can significantly decrease the number of metabolic risk factors (5). Obesity with a BMI over 35 kg/m^2^ is classified as a severe obesity disorder as there are some differences in the pathophysiology between patients with a BMI over 35 and those with a BMI between 25 and 35 kg/m^2^. Consequently, individuals with a BMI over 35 kg/m^2^ are treated and cared for differently. Based on such background, in Japan, there is a system in which additional fees can be received if registered dietitians provide nutritional guidance to patients with a BMI over 30 kg/m^2^.

Although registered dietitians are present at each hospital and public health center, there is no established guidance for measuring obesity. While it is advised that individuals reduce overall calorie intake by approximately 500–750 kcal/day or 30% of the calculated energy consumed (6–8), studies have shown the efficacy of a low-carbohydrate diet (9–11). However, initial nutritional guidance often involves an interview to evaluate dietary habits, which ends with a recommendation for an appropriate calorie intake. Therefore, it is not clear that patients with obesity, especially those who visit general hospitals for other diseases, receive proper nutritional guidance and ongoing follow-up. In Japan, patients with moderate obesity are common; however, it is possible that appropriate nutritional guidance or other interventions may not be adequately provided for patients with moderate obesity despite the existence of a system for nutritional guidance by registered dieticians for patients with a BMI over 30 kg/m^2^.

This study aimed to investigate whether nutritional guidance interventions are appropriately provided to patients with obesity. Additionally, we evaluated the effectiveness of nutritional guidance interventions in improving obesity and metabolic parameters over a short period of time. This report outlines the potential effects of nutritional guidance interventions conducted by a registered dietitian on various clinical parameters.

## 2. Materials and methods

### 2.1. Study population

This retrospective study was conducted at the Department of Nutrition of the Kawasaki Medical School General Medical Center, Japan. It involved patients with obesity with a BMI over 30 kg/m^2^ between April 2018 and March 2020. In Japan, there is a system in place that allows registered dietitians to receive additional compensation for providing nutritional guidance to patients with a BMI over 30 kg/m^2^. Therefore, we selected patients with a BMI over 30 kg/m^2^ for this study. In addition, since we provided nutritional guidance directly to each participant, we restricted our study to adults (≥20 years old) and therefore selected all patients with a BMI over 30 kg/m^2^. This study protocol was approved by the Research Ethics Committee (REC) of Kawasaki Medical School and Hospital (protocol code 3870-00). Since this study was retrospective, we provided public information about it *via* the hospital homepage instead of obtaining informed consent from each patient.

**Figure 1:**
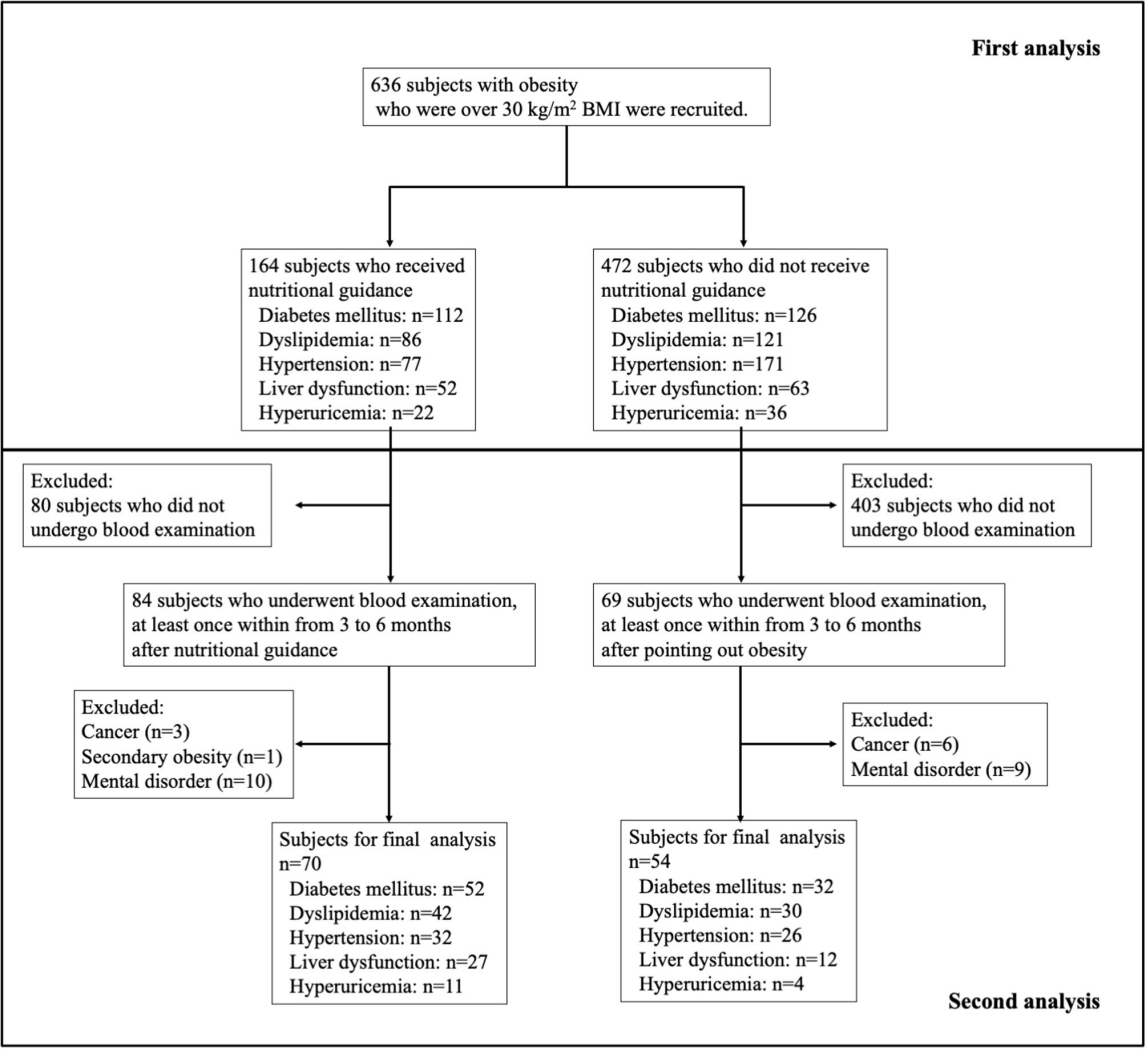
Flow chart of this study patients.

First, we recruited 636 patients with obesity who have a BMI over 30 kg/m^2^ for this study through a review of medical records. Second, in the patients with obesity (with a BMI over 30 kg/m^2^), we examined whether they received nutritional guidance from a registered dietitian. In addition, we investigated which department ordered nutritional guidance from doctors for patients with obesity (over 30 kg/m^2^ of BMI) and what percentage of patients had obesity-related diseases. This indicates that initial nutritional guidance with a registered dietitian involved, at least partially, an assessment of dietary habits through an interview and recommendations for an appropriate calorie intake. Third, we recruited 70 patients who underwent a blood examination right before receiving nutritional guidance, followed by another blood examination at least one time every 3–6 months after receiving nutritional guidance. In addition, we selected 54 patients who underwent a blood examination after being diagnosed with obesity and then another blood examination at least one time every 3–6 months after being diagnosed with obesity. We excluded patients with cancer, secondary obesity, mental disorders, and/or those using steroid drugs. Figure 1 shows the flowchart outlining the selection and exclusion criteria for patients in this study.

### 2.2. Statistical analysis

The clinical characteristics of the patients used in the analysis were age, gender, body weight and BMI, laboratory findings, and complications of obesity-associated diseases. The Mann–Whitney *U* and the chi-square tests were performed to examine the possible influence of nutritional guidance on various clinical parameters. A *p*-value >0.05 was considered statistically significant. Statistical software used was Excel Statistics for Mac version 16.54 (Social Research Information, Tokyo, Japan) and JMP, version 14.0.1 (SAS Institute Inc.).

## 3. Results

### 3.1. Names of departments ordering nutritional guidance with a registered dietitian from doctors

**Figure 2:**
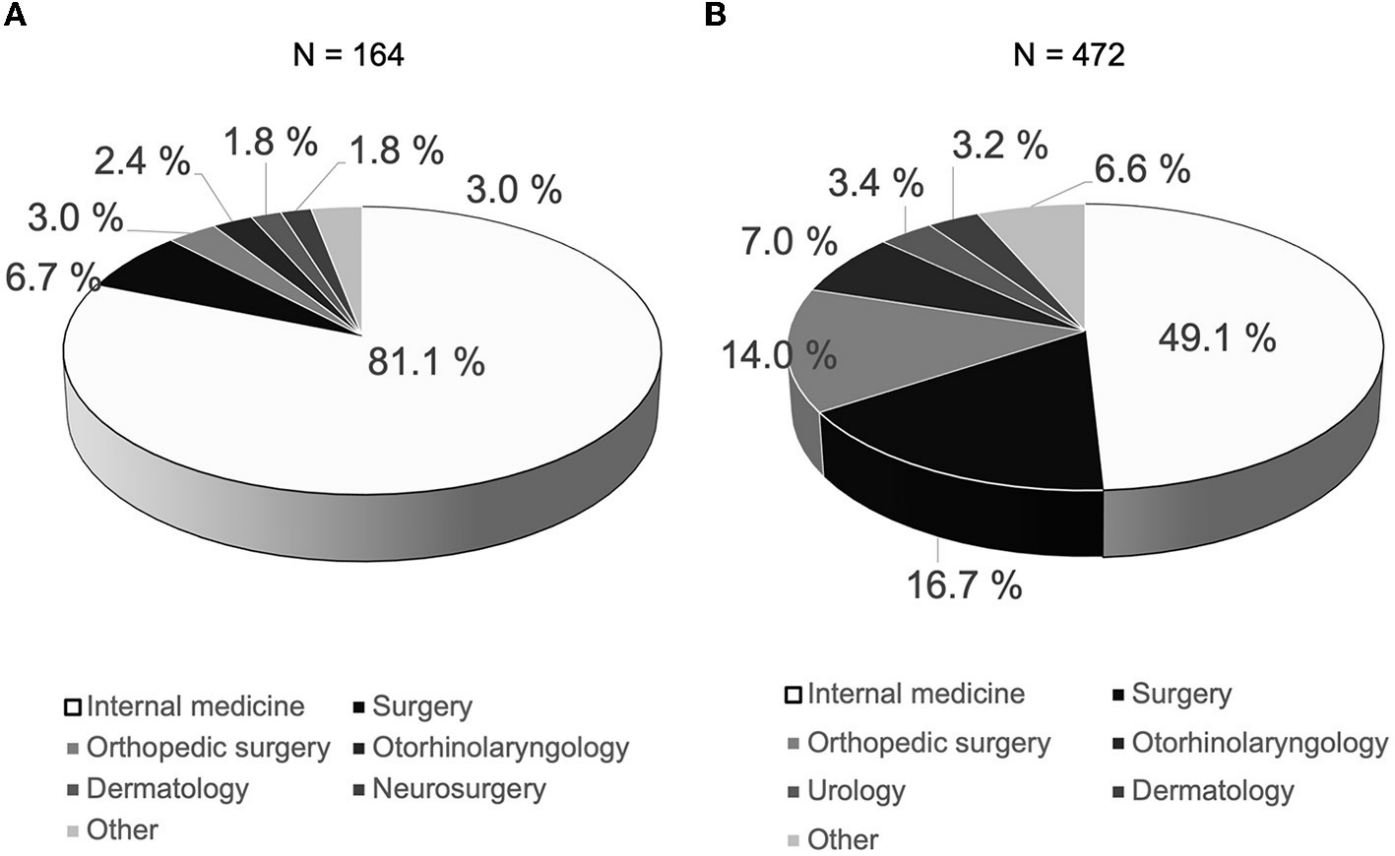
Names of department ordering nutritional guidance with registered dietitian from doctors. A total of 636 patients with obesity whose BMI were over 30 kg/m^2^ were included in this study. **(A)** A total of 164 patients with obesity whose BMI were over 30 kg/m^2^ received nutritional guidance from registered dietitian at least one time. The name of the department in which nutritional guidance was requested from doctors most frequently was internal medicine, which was followed by surgery, orthopedic surgery, otorhinolaryngology, dermatology, neurosurgery, and others doctors. **(B)** A total of 472 patients with obesity whose BMI were over 30 kg/m^2^ did not receive nutritional guidance from a registered dietitian. The name of the department in which nutritional guidance was not requested from doctors most frequently was also internal medicine, which was followed by surgery, orthopedic surgery, otorhinolaryngology, urology, dermatology, and others doctors.

The names of the departments ordering nutritional guidance with a registered dietitian from doctors are shown in Figure 2. A total of 636 patients with obesity who had a BMI over 30 kg/m^2^ were included in this study. A total of 164 patients with obesity who had a BMI over 30 kg/m^2^ received nutritional guidance from a registered dietitian at least one time, but 472 patients with obesity who had a BMI over 30 kg/m^2^ did not receive such guidance. As shown in Figure 2, most nutritional guidance with a registered dietitian was ordered from internal medicine (81.1%), which was followed by surgery (6.7%), orthopedic surgery (3.0%), otorhinolaryngology (2.4%), dermatology (1.8%), neurosurgery (1.8%), and others (3.0%). The names of the departments that did not order nutritional guidance with a registered dietitian were as follows: internal medicine (49.2%), surgery (16.7%), orthopedic surgery (14.0%), otorhinolaryngology (7.0%), urology (3.4%), dermatology (3.2%), and others (6.6%). Most of the patients who received nutritional guidance from a registered dietitian had comorbidity of lifestyle-related disease [diabetes mellitus, 68.3% (*n* = 112); dyslipidemia, 52.4% (*n* = 86); hypertension, 47.0% (*n* = 77); liver dysfunction, 31.7% (*n* = 52); and hyperuricemia, 13.4% (*n* = 22)], and we believed that much of the nutritional guidance provided by the registered dietitian was aimed at managing the aforementioned disorders (Supplementary Table 1). In contrast, the complications observed in patients who did not receive nutritional guidance were as follows: diabetes mellitus, 26.7% (*n* = 126); dyslipidemia, 23.7% (*n* = 112); hypertension, 36.2% (*n* = 171); liver dysfunction, 13.3% (*n* = 63); and hyperuricemia, 7.6% (*n* = 36).

### 3.2. The frequency of ongoing follow-up is very low in patients with obesity in general hospitals

To examine the effectiveness of nutritional guidance with a registered dietitian for patients with obesity, we recruited two groups: those who underwent a blood examination after receiving nutritional guidance from a registered dietitian and those who were diagnosed with but did not receive nutritional guidance. However, most patients who did not receive nutritional guidance did not undergo ongoing follow-up with a blood examination, even those with moderate or severe obesity. After receiving nutritional guidance from a registered dietitian or being diagnosed with obesity, we conducted blood examinations on 70 and 54 patients, respectively, at least once within a period of 3 to 6 months. Therefore, most patients with obesity did not receive ongoing follow-ups. In addition, for most patients, follow-up examinations were ordered by the Department of Internal Medicine (95.2%), including outpatients being treated by other departments. After a 3–6 month period, there was no significant difference in the prevalence of various complications or clinical parameters between patients with obesity who had a BMI over 30 kg/m^2^, regardless of whether or not they received nutritional guidance from a registered dietitian (Supplementary Table 2).

### 3.3. Characteristics of the study patients who received nutritional guidance from a registered dietitian and from those who did not

We recruited 70 patients who received nutritional guidance from a registered dietitian and 54 patients who did not receive it after a diagnosis of obesity. The clinical characteristics of the patients in this study are shown in Table 1. There were no significant differences in the various clinical characteristics (body weight, BMI, total protein, and albumin) between patients who received nutritional guidance from a registered dietitian and those who did not. Lipid metabolism markers such as total and low-density lipoprotein cholesterol levels in patients who received nutritional guidance from a registered dietitian were significantly different from those who did not. However, there was no significant difference in high-density lipoprotein cholesterol and triglycerides. There were no significant differences in glucose metabolism markers, such as plasma glucose and HbA1c levels, between patients who received nutritional guidance from a registered dietitian and from those who did not. The parameters related to liver function and kidney function in the patients who received nutritional guidance from a registered dietitian and from those who did not were not significantly different.

**Table 1:** Comparison of various parameters in patients with and without before receiving nutritional guidance with a registered dietitian.

**Clinical parameter**	**Nutritional guidance (+) *n* = 70**	**Nutritional guidance (–) *n* = 54**	***p*-value**
Age (years)	55.8 ± 14.4	57.0 ± 15.0	0.5370
Men/women	43 / 27	29 / 25	
Body weight (kg)	91.0 ± 14.5	87.0 ± 13.4	0.1364
BMI (kg/m^2^)	33.4 ± 3.4	32.6 ± 2.9	0.1883
Total protein (g/dL)	7.4 ± 0.5	7.3 ± 0.6	0.7454
Albumin (g/dL)	4.3 ± 0.4	4.3 ± 0.5	0.8879
Total cholesterol (mg/dL)	192.9 ± 37.8	176.2 ± 35.5	0.0253^*^
HDL cholesterol (mg/dL)	50.7 ± 12.6	52.3 ± 12.1	0.4027
LDL cholesterol (mg/dL)	115.9 ± 35.1	99.6 ± 30.4	0.0052^*^
Triglyceride (mg/dL)	147.9 ± 77.7	170.1 ± 92.2	0.1854
Plasma glucose (mg/dL)	150.7 ± 60.7	141.3 ± 52.2	0.4206
Hemoglobin A1c (%)	7.3 ± 1.6	7.3 ± 1.2	0.5770
AST (U/L)	36.0 ± 21.1	34.0 ± 29.2	0.1023
ALT (U/L)	46.7 ± 38.0	39.9 ± 35.1	0.1858
γ-GTP (U/L)	69.0 ± 76.3	59.5 ± 77.0	0.1146
Uric acid (mg/dL)	5.8 ± 1.4	5.6 ± 1.5	0.3650
Creatinine (mg/dL)	0.80 ± 0.30	0.75 ± 0.22	0.2013
BUN (mg/dL)	15.5 ± 5.9	15.0 ± 6.0	0.4163
Diabetes mellitus	52 (74.3%)	32 (59.3%)	
Dyslipidemia	42 (60.0%)	30 (55.6%)	
Hypertension	32 (45.7%)	26 (48.1%)	
Liver dysfunction	27 (38.6%)	12 (22.2%)	
Hyperuricemia	11 (15.7%)	4 (7.4%)	

Comparison of various values among patients in this study with and without receiving nutritional guidance from a registered dietitian. Data presented as mean ± standard deviation. ^*^p < 0.05 with Mann–Whitney U test compared to perform nutritional guidance from a registered dietitian or not. A P-value of < 0.05 was considered statistical significant.

BMI, body mass index; LDL, low-density lipoprotein; HDL, high-density lipoprotein; AST, aspartate aminotransferase; ALT, alanine aminotransferase; γ-GTP, γ-glutamyl transpeptidase; BUN, blood urea nitrogen.

### 3.4. Effect of nutritional guidance with a registered dietitian in patients with obesity

Previous studies have reported a close association between nutritional guidance and improvements in BMI or metabolic markers in Japanese patients (12). In this study, we compared the changes in blood examination results 3–6 months after the initial assessment between patients who received nutritional guidance from a registered dietitian and from those who did not (Table 2). The BMI of patients who received nutritional guidance from registered dietitians decreased compared to those who did not receive such guidance. However, dyslipidemia-associated metabolic markers were significantly decreased only among the patients who received nutritional guidance from a registered dietitian as compared to those who did not receive such guidance.

**Table 2:** Comparison of change in various clinical parameters 3–6 months later in patients with and without receiving nutritional guidance with a registered dietitian.

**Clinical parameter**	**Nutritional guidance (+) *n* = 70**	**Nutritional guidance (–) *n* = 54**	***p*-value**
Body weight (kg)	**–**0.8 ± 2.5	**–**0.3 ± 3.2	0.3689
BMI (kg/m^2^)	**–**0.3 ± 1.0	**–**0.1 ± 1.4	0.3965
Total protein (g/dL)	**–**0.1 ± 1.0	**–**0.1 ± 0.7	0.4567
Albumin (g/dL)	**–**0.0 ± 0.5	**–**0.1 ± 0.6	0.1741
Total cholesterol (mg/dL)	**–**9.7 ± 29.3	2.3 ± 22.0	0.0208^*^
HDL cholesterol (mg/dL)	**–**0.2 ± 7.5	**–**2.1 ± 15.0	0.1386
LDL cholesterol (mg/dL)	**–**10.4 ± 30.5	**–**2.0 ± 26.0	0.0147^*^
Triglyceride (mg/dL)	**–**7.1 ± 72.7	7.0 ± 91.0	0.6807
Plasma glucose (mg/dL)	**–**4.5 ± 60.8	**–**2.0 ± 51.0	0.9249
Hemoglobin A1c (%)	**–**0.3 ± 0.9	**–**0.2 ± 0.8	0.6154
AST (U/L)	**–**1.7 ± 12.1	0.3 ± 15.0	0.7182
ALT (U/L)	**–**4.1 ± 23.9	**–**0.1 ± 22.0	0.3178
γ-GTP (U/L)	**–**0.7 ± 34.3	**–**1.5 ± 20.0	0.5928
Uric acid (mg/dL)	0.0 ± 1.1	0.1 ± 1.2	0.6580
Creatinine (mg/dL)	**–**0.03 ± 0.19	0.02 ± 0.20	0.2028
BUN (mg/dL)	0.0 ± 3.8	**–**0.5 ± 4.3	0.8796

Comparison of various values among patients in this study with and without receiving nutritional guidance from a registered dietitian. Data presented as mean ± standard deviation. ^*^p < 0.05 with Mann–Whitney U test compared to perform nutritional guidance from a registered dietitian or not. A P-value of < 0.05 was considered statistical significant.

BMI, Body mass index; LDL, Low-density lipoprotein; HDL, high-density lipoprotein; AST, aspartate aminotransferase; ALT, alanine aminotransferase; γ-GTP, γ-glutamyl transpeptidase; BUN, blood urea nitrogen.

## 4. Discussion

The goal of treating patients with obesity is to reduce or prevent the risk of obesity-related diseases and health problems by targeting the reduction of body weight and visceral fat rather than solely focusing on body weight reduction. It has been reported that, in comparison to other populations, the Japanese generally have a relatively low BMI but a high proportion of visceral fat in their adipose tissue (2). In addition to obesity, an increase in the visceral fat area has also been identified as a risk factor for various obesity-related health disorders (13). In particular, the accumulation of visceral fat in Japanese individuals with moderate obesity can lead to an increased risk of various lifestyle-related diseases. Even in patients with obesity who have no underlying health disorders, the accumulation of visceral fat can be a predictor of the onset of disease. Therefore, it is recommended to diagnose obesity, measure its severity, and provide appropriate treatment. However, despite the existence of a system for nutritional guidance with a registered dietitian in general hospitals, our study's findings indicate that only a small percentage of patients with moderate obesity receive such guidance.

Moreover, this study highlighted the lack of nutritional guidance and treatment during hospitalization, except for the main diseases. In general hospitals that provide acute care treatment, attention may be paid only to the main diseases, with inadequate nutritional guidance provided for any resulting complications, especially for patients with obesity who do not exhibit any symptoms. Additionally, it is difficult to confirm whether chronic disease follow-up continues, especially after being transferred to the next hospital. Considering that patients with mild obesity in Japan are likely to develop various lifestyle-related diseases, it is possible that the absence of nutritional guidance leads to unfavorable results. In addition, another problem is that many patients with obesity are not followed up with after a diagnosis of obesity on whether they make a few and/or short-term visits to the hospital for other diseases. It is believed that the high follow-up rate in internal medicine is due to obesity-related diseases such as diabetes mellitus and dyslipidemia. Even in departments outside of internal medicine, providing nutritional guidance only after a diagnosis of obesity can have a favorable effect on patients with obesity. Therefore, patients with obesity should proactively seek and use nutritional guidance.

Another question is whether a few short-term nutritional guidance interventions are beneficial for patients with obesity. Since restricting energy intake is the most effective and established method for weight loss, dietary therapy is the basic treatment for patients with obesity (7). The results of the Japanese intervention study confirmed that even a 3% weight loss can improve health problems (12). In the present study, there was no significant difference in BMI between patients who received nutritional guidance 3–6 months later and those who did not. However, dyslipidemia-associated metabolic markers decreased significantly after the patients received nutritional guidance.

Moreover, other metabolic markers of diabetes mellitus or liver dysfunction decreased, although the change was not significant. In patients with obese diabetes mellitus, weight loss is known to decrease the risk of outcomes such as cardiovascular events (14, 15). We believe that even a few short-term nutritional guidance interventions would show favorable effects on patients with obesity, as it would make them understand the importance of nutrition.

This study has several limitations. First, this was a single-center, retrospective study. Additionally, the sample size was small, and the study involved only Japanese patients. Therefore, this study's results are not applicable to Caucasians. Second, due to a few short periods, the parameters, such as each disease, were limited to comprehending the usefulness of nutritional guidance. Third, there were no standard programs for nutritional guidance with a registered dietitian, and there was no standard verification of adherence. Fourth, the sample in this study was not homogeneous, and this sample may not necessarily be representative of the whole population. Fifth, it would be important to analyze the relationship between pathology and the number of dietary consultations and/or consider several confounding factors related to this study. We believe that the results of the current study provide an objective evaluation of how nutritional guidance interventions are provided to individuals with obesity who visit general hospitals for the management of other diseases. In addition, we described how visceral fat-associated obesity could lead to various lifestyle-related diseases. It is worth noting that a significant number of Japanese individuals with a BMI of < 25 kg/m^2^ exhibit visceral fat accumulation, suggesting that BMI may not always correlate with the accumulation of visceral fat (5). However, BMI is still used as the criteria for specific health checkups and specific health guidance aimed at identifying individuals with preliminary metabolic syndrome. Therefore, we found that a significant proportion of Japanese patients with a BMI of > 30 kg/m^2^ have accumulated visceral fat.

## 5. Conclusion

It is rare for physicians or surgeons to refer patients with obesity to a registered dietitian for nutritional guidance, especially in departments outside of internal medicine. However, diet therapy has been shown to have beneficial effects in these cases. By providing appropriate nutritional guidance, improvements in BMI and metabolic parameters can be expected. Therefore, it is important to incorporate registered dieticians in the nutritional guidance of patients with obesity as it can have a favorable influence on their health outcomes.

## Data availability statement

The original contributions presented in the study are included in the article/Supplementary material, further inquiries can be directed to the corresponding author.

## Ethics statement

This study protocol was approved by the Research Ethics Committee (REC) of Kawasaki Medical School and Hospital (protocol code 3870-00). Written informed consent for participation was not required for this study in accordance with the national legislation and the institutional requirements. Written informed consent was not obtained from the individual(s) for the publication of any potentially identifiable images or data included in this article.

## Author contributions

TA is the guarantor of this work and, as such, has full access to all data in the study and takes responsibility for the integrity of the data and its accuracy. KO and TA researched data and wrote the manuscript. NO, YK, FK, and MS researched data and contributed to the discussion. KK, HK, MT, and MS reviewed the manuscript.
