# Supplementary material for: Impact of nutritional guidance on various clinical parameters in patients with moderate obesity: A retrospective study
Source: Front Nutr. 2023 Mar 16;10:1138685. doi: 10.3389/fnut.2023.1138685 (PMC10060631; doi:10.3389/fnut.2023.1138685)
Supplement: Supplementary file 1 [file Table_1.docx]

**Supplementary Table 1.** Prevalence of various complications in subjects with obesity who were over 30

kg/m^2^ of BMI with and without receiving nutritional guidance with registered dietitian

| Complication | Nutritional guidance (+)  n = 164 | Nutritional guidance (-)  n = 472 | Chi-Square | p value |
| --- | --- | --- | --- | --- |
| Diabetes mellitus | 112 (68.3 %) | 126 (26.7 %) | 89.934 | <0.0001* |
| Dyslipidemia | 86 (52.4 %) | 112 (23.7 %) | 39.829 | <0.0001* |
| Hypertension | 77 (47.0 %) | 171 (36.2 %) | 5.8820 | 0.0153* |
| Liver dysfunction | 52 (31.7 %) | 63 (13.3 %) | 27.698 | <0.0001* |
| Hyperuricemia | 22 (13.4 %) | 36 (7.6 %) | 4.919 | 0.0266* |

Prevalence of various complications among subjects in this study with obesity who were over 30 kg/m^2^ of BMI with and without receiving nutritional guidance with registered dietitian. *p < 0.05 with Chi-Square test comparing subjects with and without receiving nutritional guidance with registered dietitian. p<0.05 was considered as statistical significance.
