# Supplementary material for: Impact of nutritional guidance on various clinical parameters in patients with moderate obesity: A retrospective study
Source: Front Nutr. 2023 Mar 16;10:1138685. doi: 10.3389/fnut.2023.1138685 (PMC10060631; doi:10.3389/fnut.2023.1138685)
Supplement: Supplementary file 2 [file Table_2.docx]

**Supplementary Table 2.** Prevalence of various complications in various clinical parameters 3-6 months

later in subjects with obesity who were over 30 kg/m^2^ of BMI with and without receiving nutritional

guidance with registered dietitian

| Complication | Nutritional guidance (+)  n = 70 | Nutritional guidance (-)  n = 54 | Chi-Square | p value |
| --- | --- | --- | --- | --- |
| Diabetes mellitus | 52 (74.3 %) | 32 (59.3 %) | 3.150 | 0.0759 |
| Dyslipidemia | 42 (60.0 %) | 30 (55.6 %) | 0.247 | 0.6190 |
| Hypertension | 32 (45.7 %) | 26 (48.2 %) | 0.073 | 0.7877 |
| Liver dysfunction | 27 (38.6 %) | 12 (22.2 %) | 3.779 | 0.0519 |
| Hyperuricemia | 11 (15.7 %) | 4 (7.4 %) | 1.978 | 0.1596 |

Prevalence of various complications among subjects in this study with obesity who were over 30 kg/m^2^ of BMI with and without receiving nutritional guidance with registered dietitian. *p < 0.05 with Chi-Square test comparing subjects with and without receiving nutritional guidance with registered dietitian. p<0.05 was considered as statistical significance.
